# Natural capital investments in China undermined by reclamation for cropland

**DOI:** 10.1038/s41559-023-02198-3

**Published:** 2023-09-25

**Authors:** Lingqiao Kong, Tong Wu, Yi Xiao, Weihua Xu, Xiaobiao Zhang, Gretchen C. Daily, Zhiyun Ouyang

**Affiliations:** 1 State Key Laboratory of Urban and Regional Ecology, Research Center for Eco-Environmental Sciences, Chinese Academy of Sciences, Beijing, China; 2 Natural Capital Project, Stanford University, Stanford, CA USA; 3 Department of Biology and Woods Institute for the Environment, Stanford University, Stanford, CA USA

**Keywords:** Earth and environmental sciences, Ecology

## Abstract

Globally, rising food demand has caused widespread biodiversity and ecosystem services loss, prompting growing efforts in ecological protection and restoration. However, these efforts have been significantly undercut by further reclamation for cropland. Focusing on China, the world’s largest grain producer, we found that at the national level from 2000 to 2015, reclamation for cropland undermined gains in wildlife habitat and the ecosystem services of water retention, sandstorm prevention, carbon sequestration and soil retention by 113.8%, 63.4%, 52.5%, 29.0% and 10.2%, respectively. To achieve global sustainability goals, conflicts between inefficient reclamation for cropland and natural capital investment need to be alleviated.

## Abstract (web summary)

Between 2000 and 2015, reclamation for cropland in China undermined gains in wildlife habitat and the ecosystem services of water retention, sandstorm prevention, carbon sequestration and soil retention by 113.8%, 63.4%, 52.5%, 29.0% and 10.2%, respectively.

## Main

Population growth, rising food demand and rapid, large-scale urbanization have driven cropland encroachment of natural ecosystems across the world, leading to profound losses of biodiversity and terrestrial ecosystem services^1–3^. Moreover, the shift of cropping systems to marginal lands is causing further negative impacts^4,5^. Growing concerns about these impacts have prompted the worldwide implementation of ecological restoration programmes to increase the provision of ecosystem services for human well-being^6^.

Since the year 2000, China has made substantial, world-leading investments in mitigating land degradation and restoring ecosystem services^7,8^. This has occurred through large-scale transformations of cropland into grasslands and forests, which have led to significant improvements in important ecosystem services^7,9,10^. However, coming from the other direction, extensive reclamation for cropland has caused grave losses in these same services—a fact not well recognized or quantified in the existing literature. Here, we temporally and spatially assess the impacts of reclamation for cropland on vital ecosystems and their services in China, the world’s most populous and largest grain-producing country, from 2000 to 2015. This study shows the extent and pattern of cropland reclamation-induced reversals in natural capital investment at the national level.

Based on data for the years 2000–2015 from the national ecosystem assessments, we mapped the biophysical supply of four key ecosystem regulating services—water retention, soil retention, sandstorm prevention and carbon sequestration—as well as potential habitat for wildlife, at a spatial resolution of 90 × 90 m^2^ (refs. ^9,11^). We then quantified the relative importance of each pixel in regard to supply of ecosystem services using an integrated index that classifies all pixels into one of four levels of importance: ‘vital’, ‘important’, ‘moderate’ and ‘general’ (ref. ^9^). This was done to identify the importance of ecosystems that had been reclaimed into cropland. We obtained the loss–gain ratio of each ecosystem service and analysed the different types of reclamation-induced change based on climatic conditions, terrain, soil, food production and ecosystem service supply.

## Results

### Loss of vital ecosystems by reclamation for cropland

**Fig. 1:**
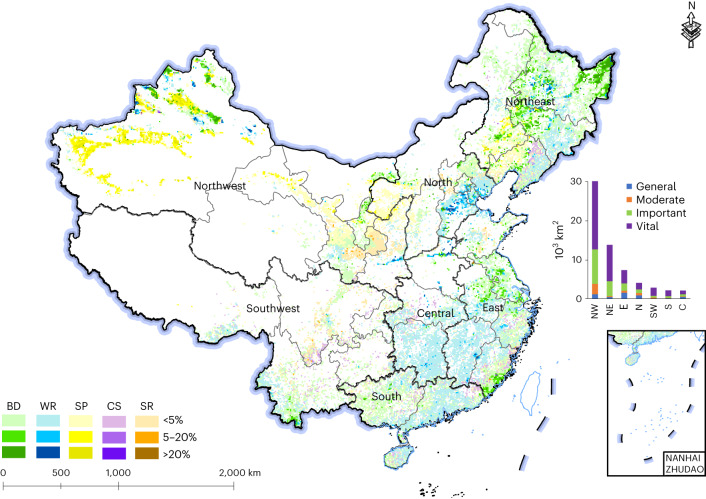
Vital ecosystems converted to cropland across China, 2000–2015. Colours represent the dominant ecosystem services (or biodiversity) of a given area (10 × 10 km^2^ plot): the darker the colour, the higher the proportion of vital ecosystems converted to cropland. The histogram on the right shows the areal composition of ecosystems of varying importance levels that were reclaimed. BD, biodiversity; WR, water retention; SP, sandstorm prevention; CS, carbon sequestration; SR, soil retention.

Over the study period, 21,215 km^2^ of forest and shrub, 25,579 km^2^ of grassland and 12,278 km^2^ of wetland were converted to cropland in China. We found that loss of natural ecosystems was concentrated in those areas most critical in regard to ecosystem regulating services and biodiversity, with vital and important ecosystems, respectively, accounting for 58.1% and 26.8% of the total reclaimed area (Fig. 1) (for ecosystem importance classification definitions, see Methods and Supplementary Table 1).

Reclamation for cropland was most intense in the ecologically vital or fragile areas of northern, eastern and southern China, including the Sanjiang Plains, Songnen Plains, Tarim Basin, Junggar Basin, the hills of southern Fujian and the lower reaches of the Yellow River Basin. Ecologically important regions that received substantial ecosystem protection and restoration investments over the past two decades, but where reclamation for cropland was also widespread and scattered, included the Changbai Mountain, Loess Plateau, Wuling Mountains, the tropical forests of southern Yunnan and China’s southeast coastal wetlands. Across these regions, reclamation for cropland significantly undercut gains in wildlife habitat, water retention, sandstorm prevention, carbon sequestration and soil retention. For example, the Sanjiang and Songnen Plains are key areas in regard to China’s food production (especially cereal crops), but they also have rich wetland ecosystems that are important habitats for migratory birds.

### Undermining of wildlife habitat and ecosystem services

**Fig. 2:**
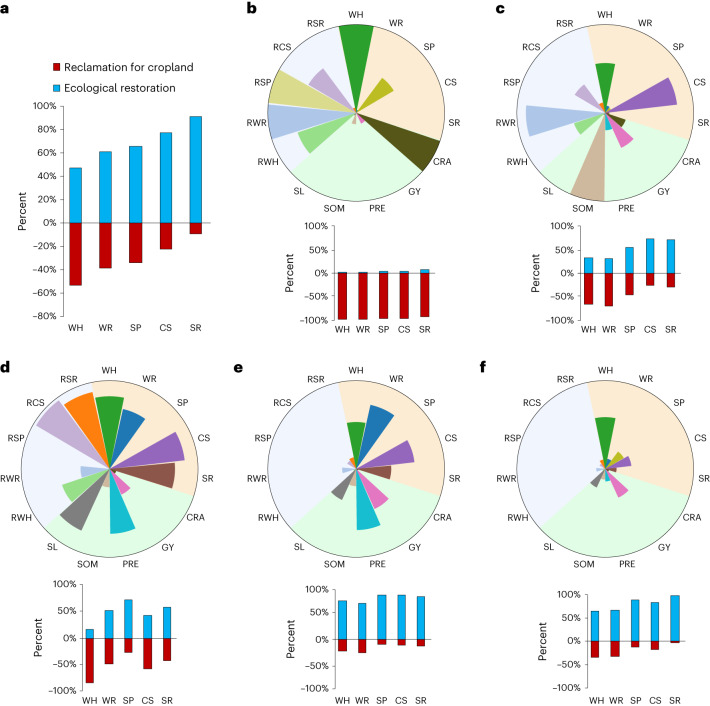
Gains in primary ecosystem regulating services and wildlife habitat due to ecosystem protection and restoration were undercut by reclamation for cropland across China from 2000 to 2015. **a**–**f**, Blue indicates gains from ecosystem protection and restoration, and red indicates losses from reclamation for cropland. **a**, Total gains and losses for the whole country. **b**–**f**, Rosette diagrams of five clusters of reclamation-induced ecosystem service losses at the provincial level. **b**, the first cluster, representing areas experienced the largest loss of restoration gains in ecosystem services; **c**, the second cluster, representing areas providing a large supply of carbon sequestration, wildlife habitat and high grain yield, but that experienced large losses in biodiversity and ecosystem services; **d**, the third cluster, representing areas providing high ecosystem regulating services and biodiversity, with a humid climate and high slopes for newly reclaimed land, experienced large losses of ecosystem services; **e**, the fourth cluster, representing areas underwent relatively low loss of biodiversity and ecosystem services due to ecological restoration, with a humid climate; **f**, the fifth cluster, representing areas underwent low loss of biodiversity and ecosystem service. The histogram below each rosette summarizes gains and losses for each cluster. WH, proportion of wildlife habitat; WR, capacity for water retention; SP, capacity for sandstorm prevention; CS, capacity for carbon sequestration; SR, capacity for soil retention; RWH, reduction in wildlife habitat due to reclamation; RWR, reduction in water retention due to reclamation; RSP, reduction in sandstorm prevention due to reclamation; RCS, reduction in carbon sequestration due to reclamation; RSR, reduction in soil retention due to reclamation; GY, grain yield; CRA, area of reclamation for cropland; PRE, annual precipitation; SL, average slope; SOM, soil organic matter content.

At the national level, we quantified the loss of ecosystem services due to reclamation for cropland as reversal of restoration-related gains from 2000 to 2015. Over these 15 years, 25,138 km^2^ of potential habitat for nationally protected plants and animals, 3.0 billion m^3^ of water retention capacity, 28.0 million tons of sandstorm prevention capacity, 18.1 million tons of carbon sequestration capacity and 19.4 million tons of soil retention capacity were lost. As a percentage of the gains in these ecosystem services from ecological restoration efforts over the same period, the corresponding losses were 113.8%, 63.4%, 52.5%, 29.0% and 10.2% (Fig. 2).

These counteracting losses due to reclamation for cropland correspond to three primary area types: (1) areas providing a large portion of the sandstorm prevention service and wildlife habitat, but also with an arid climate, low soil organic matter content and low grain yield; these areas experienced the largest loss of restoration gains in ecosystem services and were typically in marginal lands in northwestern China (Fig. 2b); (2) areas providing a large supply of carbon sequestration and wildlife habitat, with a relatively arid climate and high soil organic matter content in newly reclaimed land and boasting high grain yield, but that experienced large losses in biodiversity, water retention and carbon sequestration; these areas were located primarily in the wetlands of northeastern China (Fig. 2c); and (3) areas providing large supplies of primary ecosystem regulating services and wildlife habitat, with a humid climate and high slopes for newly reclaimed land; these areas experienced large losses of ecosystem services and were primarily located in the mountainous terrain of southern and southwestern China, which also covers biodiversity hotspots (Fig. 2d). The remaining areas (Fig. 2e,f) underwent relatively low loss of biodiversity and ecosystem services due to ecological restoration. However, as a general pattern, we found that impacts were greater where the supply of ecosystem services was greater and the climate wetter, as well as with increasing slope of newly reclaimed cropland (corresponding mainly to areas in eastern and southern China; Fig. 2e).

China’s total crop production increased by 34.5% between 2000 and 2015, but we found that newly reclaimed cropland contributed only 9.8% to the total increase. This shows that the increase in crop production during this period was primarily due to improved production efficiency rather than cropland expansion. Most new croplands were of poor quality and were less suitable for crop production^5^, while most of the cropland lost was of high productivity. For example, there has been a substantial decline in the quality of newly reclaimed cropland in northern China^12^.

**Extended Data Fig. 1:**
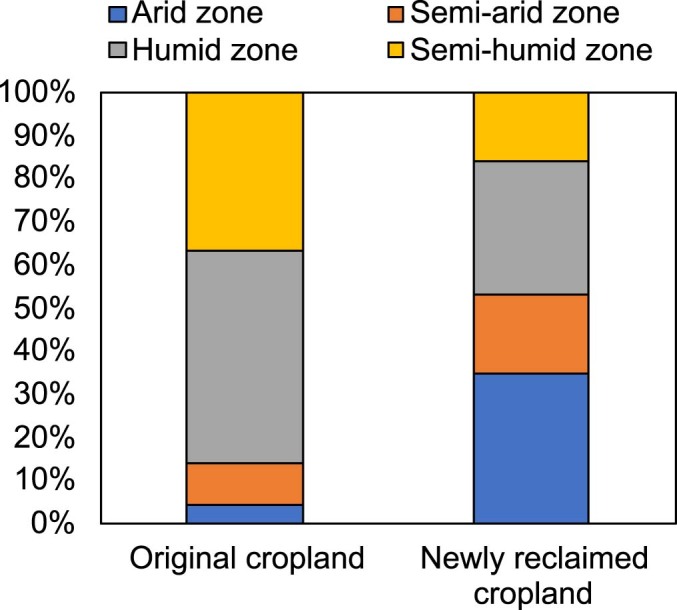
Proportions of original cropland and newly reclaimed cropland in arid and humid zones. The proportions of original cropland from 2000 and those of newly reclaimed cropland between 2000 and 2015 in different arid and humid zones of China were analyzed.

**Extended Data Fig. 2:**
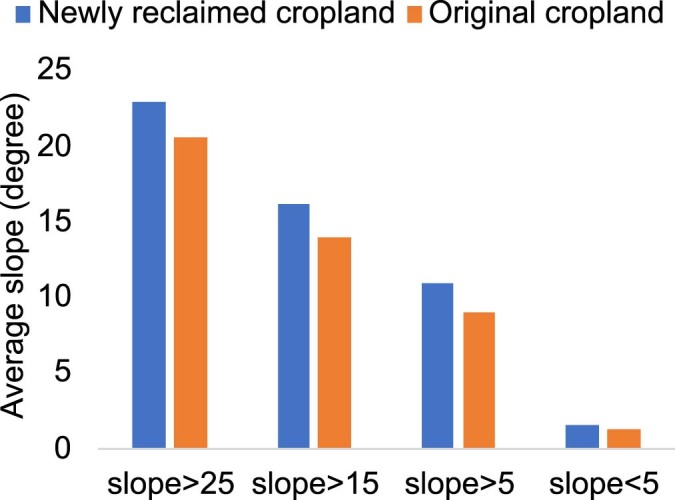
Slope levels of original cropland and newly reclaimed cropland. The average slope levels of original cropland from 2000 and those of newly reclaimed cropland between 2000 and 2015 were analyzed.

**Extended Data Fig. 3:**
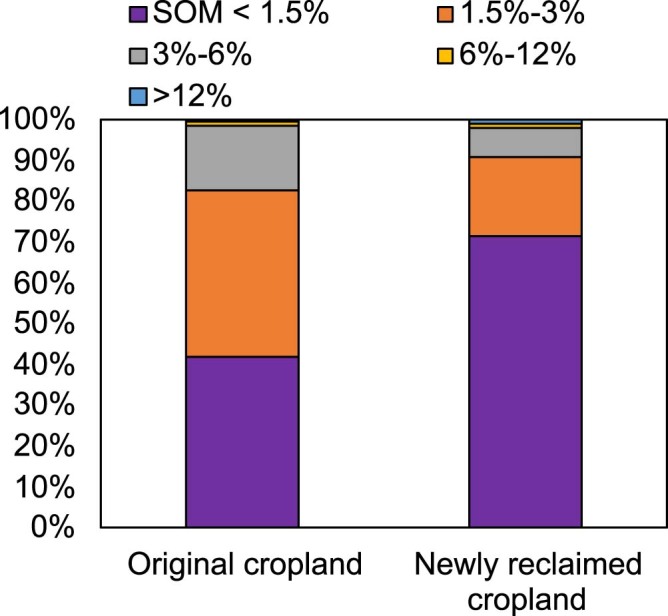
Proportions of original cropland and newly reclaimed cropland in different levels of soil organic matter content. The proportions of original cropland from 2000 and those of newly reclaimed cropland between 2000 and 2015 in different levels of soil organic matter content were analyzed.

The economic inefficacy of reclamation for cropland in China can, in part, be attributed to biophysical and geographic constraints, which will become more restrictive with climate change. Climatic, topographical and soil fertility conditions determine a given landscape’s suitability for cultivation, and we found that recent reclamation for cropland in China had mostly occurred in locations where such factors militate against expansion. While >85% of existing cropland was distributed in humid and semihumid areas, new croplands from 2000 to 2015 were mostly (52.2%) distributed in arid and semi-arid areas, of which 65.6% was in arid areas (Extended Data Fig. 1). In particular, adverse climatic conditions greatly restricted the productivity of newly reclaimed cropland in northern China^12^. Additionally, reclamation during the study period expanded over land that had, on average, 21.0% higher slope than existing cropland (Extended Data Fig. 2). This reduces water retention capacity and increases the risk of soil erosion, flooding and disastrous landslides. Finally, the soil fertility of newly reclaimed cropland tended to be much lower than that of existing cropland: the proportion of land with <1.5% soil organic matter was 70% greater (Extended Data Fig. 3).

## Discussion

This study has shown that efforts made in ecological protection and restoration have been significantly undermined by further reclamation for cropland. In the coming decades, secular trends of income growth and urbanization will continue to drive rising food demand, both in China and across the world^1^. This will increase pressures on crop production to meet food security concerns. If inefficient reclamation for cropland continues, its negative ecological impacts will significantly undercut returns from natural capital investment and exert a further drag on agricultural production^2,3^. Furthermore, these impacts will gradually worsen over time. Strict measures should be taken to protect vital ecosystems, increase the ecological restoration of croplands in marginal and fragile landscapes and curb inefficient agricultural expansion by transitioning to sustainable intensification. To effect this transition, key policies could include these presented below.

### Identification of crucial regions for ecosystem services

More precise spatial analysis of the distribution of major ecosystem services, such as provision of wildlife habitat, water retention, sandstorm prevention, carbon sequestration and soil retention, is needed to better identify areas for ecosystem protection and restoration. This can then inform land-use policies such as China’s ecological protection redlines, national parks and nature reserves, all of which aim to rigorously protect vital ecosystems from reclamation for cropland and other deleterious forms of development^11^.

### Ecosystem restoration of croplands in marginal, biodiverse and fragile landscapes

Ecosystem restoration should target existing marginal croplands, particularly in areas with important ecosystem services, and in fragile landscapes, which include places with (1) endangered and endemic species; (2) water supply sources; (3) wetlands serving as migratory bird habitats; (4) sloping lands at risk from erosion; and (5) desertified land that could be the source of sandstorms. Biodiverse areas suitable for planting crops should be strictly protected and those that have already been reclaimed—such as the Sanjiang and Songnen Plains, plains in the middle and lower Yangtze River Basin and coastal wetlands—should be focal points for the protection and restoration of wildlife habitat. More generally, large-scale initiatives such as the Grain-to-Green programme should be enhanced and further incorporated into national programmes. For instance, the new Key Protection and Restoration Project for Major Ecosystems aims to improve ecosystem quality and service provision in degraded lands over the period 2021–2035, with investments totalling over US$400 billion^13,14^.

### Increasing crop yields through sustainable intensification

The world’s agricultural biomass can more than meet future food demand without additional cropland expansion, through higher cropping intensity^15^. Rather than reclaiming fragile and biodiverse areas, there is great potential to increase production through sustainable intensification^16^. This could include crop variety improvement^17^, irrigation system upgrades, soil fertility improvement and better field management practices for tillage regimes and crop rotation. For example, yields of rice, maize and wheat increased by 9.9%, 28.2% and 44.3%, respectively, in China from 2000 to 2015 due to such advances. Finally, high-quality cropland, especially for grain production, must be better protected from urbanization and industrialization. All efforts should be taken to break the connection between reclamation for cropland and natural ecosystem conversion.

Modern agriculture is among the primary drivers of wildlife habitat loss, environmental pollution and climate change. More than 50 years after the ‘green revolution’ enabled a major increase in food production worldwide, it is time to undergo another transformation of agriculture: an ecological revolution to harmonize food production with the protection and enhancement of biodiversity and other ecosystem services. Despite the negative consequences of reclamation for cropland globally, there have also been many examples of adaptive management achieving ‘win-win’ outcomes in China and elsewhere. For instance, natural rubber production and improvemens in key ecosystem services such as water retention, soil retention and carbon sequestration have been realized through sustainable intensification^18^. Other studies have found that food production and carbon sequestration can be jointly promoted in croplands through soil quality improvements and related management practices^19,20^. Additionally, natural forests in agricultural landscapes can benefit biodiversity maintenance while improving crop production by up to 20% (refs. ^21,22^). Nonetheless, intensification should be undertaken carefully to ensure its sustainability. Studies have found that, without proper attention to underlying ecological processes, increasing cropping intensities can put biodiversity at significant risk^2,23^.

The challenge of balancing ecological conservation and agricultural production will probably become more acute as China places greater emphasis on food security. Similar trends can be found in other countries, including advanced economies with ample arable land such as the United States^4^. What this suggests about global trends in the relationship between agricultural production and ecological conservation, and between food security and environmental sustainability, merits further research.

## Methods

### Assessments of ecosystem service and biodiversity changes

We estimated the biophysical supply of four key ecosystem regulating services—water retention, soil retention, sandstorm prevention and carbon sequestration—in China in 2000 and 2015. The data came from the national ecosystem assessments in the years 2000–2015. Water retention (soil retention, sandstorm prevention) refers to water (soil and sand) retained in ecosystems within a certain period (1 year in the case of this study).

Water retention was estimated using the water balance equation. In this model, the capacity of water retention is the difference between the amount of precipitation and the sum of runoff and evapotranspiration. Soil retention was measured by the universal soil loss equation, indicating the difference between potential and actual soil erosion in ecosystems. The sandstorm prevention service was mapped using the revised wind erosion equation. Carbon sequestration refers to carbon sequestered by terrestrial ecosystems. By examining the dynamics of biomass carbon storage in China’s forest, grassland and wetland ecosystems, average annual carbon sequestration was estimated. The detailed methods for estimation of ecosystem services analysed here were taken from Ouyang et al.^9^, and are detailed in the Methods section of Supplementary Information. Supplementary Table 2 shows the data sources for parameters.

Wildlife habitat supports biodiversity, and here we quantify the potential of habitat to support biodiversity and their relative importance. We selected threatened species from the IUCN Red List or China’s Red List as indicator species, including categories of critically endangered, endangered and vulnerable species. The list finally selected contains a total of 1,534 species, including 955 plants, 152 mammals, 127 birds, 177 amphibians and 123 reptiles. First, we collected information on their geographic distribution and then refined the potential habitat for each species based on specific distribution area, elevational range and vegetation. To quantify the relative importance of wildlife habitat, we set different weights based on the endangered level of the species and summed weighted potential habitats for each taxon. We normalized summed values separately using the minimum–maximum normalization method. We used the maximum value of each pixel among the five taxon layers to generate the overall importance index map for habitat. The detailed methods and data sources used in this part can be found in the study by Xu et al.^11^ and are detailed in the Methods section of Supplementary Information.

We estimated the losses in each ecosystem regulating service capacity (ES_l_) and wildlife habitat (WH_l_), where natural ecosystems (that is, forest, shrubland, grassland and wetland) were converted to cropland, as well as the gains in each ecosystem regulating service capacity (ES_g_) and wildlife habitat (WH_g_) where cropland was converted to natural ecosystems. The loss–gain ratios of each ecosystem regulating service (${\mathrm{lg}{\_{\mathrm{r}}}}_{{{\mathrm{ES}}}}$) and wildlife habitat (${\mathrm{lg}{\_{\mathrm{r}}}}_{{{\mathrm{WH}}}}$) due to reclamation for cropland were calculated, respectively, as follows:

$${\mathrm{lg}{\rm{\_}}{\mathrm{r}}}_{{{\mathrm{ES}}}}=\frac{{{{\mathrm{ES}}}}_{{\mathrm{l}}}}{{{{\mathrm{ES}}}}_{{\mathrm{g}}}}\times 100 \%$$

and

$${\mathrm{lg}{\rm{\_}}{\mathrm{r}}}_{{{\mathrm{WH}}}}=\frac{{{{\mathrm{WH}}}}_{{\mathrm{l}}}}{{{{\mathrm{WH}}}}_{{\mathrm{g}}}}\times 100 \%.$$

Land-cover classification images of China at 30 m resolution for 2000 and 2015 (ref. ^24^) were used to identify different ecosystem types. Classification images were derived from the environment and disaster monitoring and forecasting images of small satellite constellation (HJ-1A/B) and Landsat OLI (resolution 30 m), then through object-oriented multiscale segmentation and decision tree procedures supported by a classification sample database. Land cover was categorized into forest, shrubland, grassland, wetland, cropland, urban land, desert and bare land. The ecosystem classification system used in this study is shown in Supplementary Table 3. A total of 118,316 independent ground survey samples obtained through random sampling were used for data accuracy verification. The average accuracy of the eight first-level categories was 93.6%, and that of of the 42 third-level categories was 87.7%.

To reveal the clustering patterns of multifactorial characteristics of the negative impacts of reclamation for cropland, we selected 15 variables. These reflected reclamation extent and impacts on ecosystem services and biodiversity, regional ecological conditions, climate, soil and terrain conditions and food production efficiency for multivariate cluster analysis. The input data included: (1) the capacities for water retention, soil retention, sandstorm prevention and carbon sequestration; (2) the proportion of wildlife habitat; (3) grain yield in the year 2015; (4) annual precipitation, average slope, average soil organic matter content and area of newly reclaimed cropland between 2000 and 2015; and (5) corresponding declines in the five ecosystem services between 2000 and 2015.

Incorporating the acquisition unit of grain yield data, *k*-means cluster analysis was performed at the provincial scale. Input factors were processed by minimum–maximum normalization to normalize data. The elbow method was used to determine cluster number. We used rosette diagrams to represent the variables for each cluster and calculated the total loss–gain ratio for the corresponding provinces of each type, revealing differences in typical types under varied ecological and agricultural conditions. The loss–gain ratio of each cluster was calculated as follows:

$${\mathrm{lg}{\rm{\_}}{\mathrm{r}}}_{i{\rm{\_}}{{\mathrm{ES}}}}=\frac{{\sum }_{j=1}^{n}{{{\mathrm{ES}}}}_{{{\mathrm{l}}j}}}{{\sum }_{j=1}^{n}{{{\mathrm{ES}}}}_{{{\mathrm{g}}\,j}}}\times 100 \%$$

and

$${\mathrm{lg}{\rm{\_}}{\mathrm{r}}}_{i{\rm{\_}}{{\mathrm{WH}}}}=\frac{{\sum }_{j=1}^{n}{{{\mathrm{WH}}}}_{{{\mathrm{l}}j}}}{{\sum }_{j=1}^{n}{{{\mathrm{WH}}}}_{{{\mathrm{g}}\,j}}}\times 100 \%$$

where ${\mathrm{lg}{\_{\mathrm{r}}}}_{{i\_{\mathrm{ES}}}}$ and ${\mathrm{lg}{\_{\mathrm{r}}}}_{{i\_{\mathrm{WH}}}}$ are, respectively, the loss–gain ratios of each regulating service and wildlife habitat of cluster *i*; ${{{\mathrm{ES}}}}_{{{\mathrm{l}}j}}$ and ${{{\mathrm{WH}}}}_{{{\mathrm{l}}j}}$ are, respectively, the losses of each ecosystem regulating service capacity and wildlife habitat where natural ecosystems (that is, forest, shrubland, grassland and wetland) were converted to cropland in province *j* of cluster *i*; and ${{{\mathrm{ES}}}}_{{{\mathrm{g}}\,j}}$ and ${{{\mathrm{WH}}}}_{{{\mathrm{g}}\,j}}$ are, respectively, the increases in each ecosystem regulating service capacity and wildlife habitat where cropland was converted to natural ecosystems.

### Assessment of ecosystem service importance

We used an integrated index proposed by Ouyang et al.^9^ to quantify the relative importance of each pixel in regard to provision of ecosystem services and biodiversity. First, we ranked the importance of each pixel regarding the provision of a single service, service by service. For example, to identify critical areas for soil retention we classified all pixels into one of four levels of importance: vital, important, moderate and general. The classification procedures were: (1) calculate the soil retention of each pixel; (2) sort all pixels by soil retention capacity in descending order and then calculate the cumulative proportion of soil retention across pixels; (3) assign vital to those pixels with cumulative proportion ~0–50%, important to those with cumulative proportion ~50–75%, moderate to those with cumulative proportion ~75–90% and general to those with cumulative proportion ~90–100%.

Thereafter, the importance of each service and wildlife habitat provision was synthesized into the integrated index of ecosystem importance. We applied the maximum value method whereby the index value equals the highest importance value of any service in each pixel. Thus, a pixel was scored important if it was important for any single service, in accordance with the assumption that this indicates the irreplaceability of each ecosystem service.

### Intensity of reclamation for cropland on vital ecosystems

To understand how intensely reclamation for cropland impacts natural ecosystems of varying importance level, we first assessed the importance of ecosystem services and biodiversity across China in the year 2000 and then calculated the proportion of cropland encroachment on ecosystems for all importance levels from 2000 to 2015 according to the following formula:

$${P}_{i}=\frac{{\sum }_{j=1}^{n}{X}_{{ij}}\times {A}_{j}}{{A_{\mathrm{r}}}}\times 100 \%$$

where *P*_*i*_ is the proportion of cropland encroaching on ecosystems of importance level *i* (*i* = 1 (general), 2 (moderate), 3 (important), 4 (vital)). *A*_*j*_ is the area of the pixel *j*. If the importance level of the pixel was level *i* and it was converted from natural ecosystem to cropland between 2000 and 2015, we set *X*_*ij*_ to 1, otherwise it was set to 0. *A*_r_ is the total area of newly reclaimed cropland in China.

To spatialize the intensity of reclamation for cropland on vital ecosystems, we divided the whole country into grids of 10 × 10 km^2^ cells, calculated the proportion of vital ecosystems converted to cropland in each grid cell and then spatialized this proportion. For each grid cell we chose the ecosystem service with the largest vital area as the leading service (for example, soil retention, water retention, sandstorm prevention, carbon sequestration or biodiversity).

### Crop production and its changes

China’s provincial-level crop production data for 2000 and 2015 were taken from the China Rural Statistical Yearbook and the China Agriculture Yearbook (Supplementary Fig. 1), including those for rice, wheat, corn, soybeans and potatoes, among others. Crop yield was calculated using the following equation:

$${{{\mathrm{GYPUA}}}}_{i}=\frac{{{\rm{TGY}}}_{i}}{{{{\mathrm{Ac}}}}_{i}}$$

where GYPUA_*i*_ represents the crop yield of province *i*, TGY_*i*_ represents the total crop production of province *i* and Ac_*i*_ represents the area of cropland in province *i*. We calculated the increase in crop production due to reclamation for cropland using the following equation:

$${{{\mathrm{IGYR}}}}_{i}=\,{{{\mathrm{GYPU}}A}}_{i2015}\times {A}_{{{\mathrm{NTC}}i}}$$

where IGYR_*i*_ represents the increase in crop production due to reclamation for cropland in province *i*, ${{{\mathrm{GYPU}}A}}_{i2015}$ represents the crop yield of province *i* in 2015 and ${A}_{{{\mathrm{NTC}}i}}$ represents the area of natural ecosystems converted to cropland between 2000 and 2015 in province *i*.

We then estimated the contribution rate of reclamation for cropland to crop production increase using the following equation:

$${R}_{{\mathrm{c}}}=\frac{{\sum }_{i=1}^{n}{{{\mathrm{IGY}}R}}_{i}}{{\sum }_{i=1}^{n}({{{\mathrm{TG}}}{{\mathrm{Y}}}_{2015}}_{i}-{{{\mathrm{TG}}}{{\mathrm{Y}}}_{2000}}_{i})}\times 100 \%$$

where *R*_c_ represents the contribution rate of reclamation to crop production increase and ${{{\mathrm{TGY}}}}_{{2000}_{i}}$ and ${{{\mathrm{TGY}}}}_{{2015}_{i}}$ represent total crop production in 2000 and 2015, respectively, in province *i*; *n* represents the number of provinces.

### Suitability of newly reclaimed cropland

We compared the suitability for planting crops in newly reclaimed cropland between the years 2000 to 2015 and in the original croplands of 2000 based on: (1) the distribution of wet and dry zones in China (Supplementary Fig. 2), namely arid, semi-arid, humid and semihumid zones; for each zone we calculated the areas and proportions of original and newly reclaimed cropland (Extended Data Fig. 1). (2) We then calculated the average slope for both original and newly reclaimed cropland across the whole country and in areas with different slope ranges (Extended Data Fig. 2 and Supplementary Fig. 3). (3) We calculated the areas and proportions of original and newly reclaimed cropland with different ranges of soil organic matter content (Extended Data Fig. 3 and Supplementary Fig. 4).

### Reporting summary

Further information on research design is available in the Nature Portfolio Reporting Summary linked to this article.

### Supplementary information

Supplementary Information Supplementary methods, Figs. 1–4 and Tables 1–5.

Reporting Summary

## Data Availability

Data that support the findings of this study are available at the Science Data Bank (10.57760/sciencedb.09940).
