# Supplementary material for: Natural capital investments in China undermined by reclamation for cropland
Source: Nat Ecol Evol. 2023 Sep 25;7(11):1771–7. doi: 10.1038/s41559-023-02198-3 (PMC10627817; doi:10.1038/s41559-023-02198-3)
Supplement: Supplementary file 1 — Supplementary methods, Figs. 1–4 and Tables 1–5. [file 41559_2023_2198_MOESM1_ESM.pdf]

nature ecology & evolution

Brief Communication

https://doi.org/10.1038/s41559-023-02198-3

Natural capital investments in China undermined by reclamation for cropland

In the format provided by the authors and unedited

Supplementary Materials

Materials and Methods

Biophysical supply of ecosystem services

We used the models from China's previously-published national ecosystem assessments *(1)* to estimate the biophysical supply of ecosystem services.

(i) Soil retention

We estimated the annual amount of soil retained by ecosystems using the Universal Soil Loss Equation (USLE) *(2)* and the InVEST platform *(3, 4)*; the model can be expressed as:

$$SC = R \times K \times LS \times (1 - C)$$

where SC represents the soil retention capacity (t ha^{-1} a^{-1}), R is the rainfall erosivity factor (MJ mm ha^{-1} h^{-1} a^{-1}), K is the soil erodibility factor (t ha h ha^{-1} MJ^{-1} mm^{-1}), LS is the topographic factor and C is the vegetation cover factor.

Rainfall erosivity reflects the potential for raindrops and runoff to induce soil erosion *(5)*. In this study, we adopted the Daily Rainfall Erosivity Model *(6)*, for which only conventional rainfall data (daily precipitation) is needed.

Soil erodibility reflects the sensitivity of soil particles to erosive forces and is an internal factor affecting soil erosion that is closely related to soil attributes *(5)*. The Erosion/Productivity Impact Calculator (EPIC) was employed to calculate K using soil clay, silt, sand and organic carbon content *(7, 8)*.

The topographic factor reflects the effects of terrain (slope length and gradient) on soil erosion *(9)*. We integrated relevant research on gentle slopes and steep slopes, and performed calculations using different slope segments *(10-12)*.

The vegetation cover factor describes the effect of vegetation on soil erosion and is related to vegetation structure and cover. Different ecosystem types and vegetation

coverage were considered *(12)*. The types of ecosystems include: forests, shrubs, grasslands, wetlands, farmlands (which include paddy fields, dry lands, arbor gardens and shrub gardens). Different ecosystem types are given different values according to vegetation coverage *(13-15)*.

(ii) Sand storm prevention

We estimated the annual amount of sand retained in an ecosystem using the Revised Wind Erosion Equation (RWEQ) model *(16)*.

The RWEQ combines empirical and process modeling and has been extensively tested under broad field conditions. The RWEQ model estimates sand / soil loss at a specific point (S_L; kg m^{-2}) as a function of several factors: weather (WF); soil erodibility (EF); soil crust (SCF); surface roughness (K'); and vegetation cover (C), which permit estimation of the maximum transport capacity by wind (Q max) as follows:

$$Q_{max} = 109.8 \cdot [WF \times EF \times SCF \times K' \times C]$$

$$S = 150.71 \cdot (WF \times EF \times SCF \times K' \times C)^{-0.3711}$$

$$S_L = \frac{2 \cdot z}{S^2} Q_{MAX} \cdot e^{-(z/s)^2}$$

where Q_{max} (kg m^{-1}) is the maximum transport capacity, S (m) is the critical field length, Z (m) is the distance from the upwind edge of the field, S_L (kg m^{-2}) is the soil loss caused by wind erosion.

Weather Factor (WF) represents the influence of climate condition on wind erosion, and WF is partitioned according to the preponderance and positive parallel ratio values from the weather file *(17, 18)*. WF is determined by dividing the total wind value for each period by 500 and multiplying by the number of days in the period *(19, 20)*.

Soil Erodible Factor (EF) is that fraction of the surface 25 mm of sand / soil that is smaller than 0.84mm in diameter, as determined by a standard compact rotary sieve *(21)*. From a soil sieving data base, the highest value for EF during a year for each site was correlated with basic soil physical and chemical properties *(22)*.

Soil Crusting Factor (SCF): when raindrops impact the soil surface, there is a redistribution of soil particles and a formation of surface crust. The resulting soil

surface can be extremely hard or very fragile and may decrease or increase wind erosion potential *(23)*. The SCF equation was developed using laboratory wind tunnel tests on the resistance of soil aggregates and crusts to windblown sand *(24)*.

Vegetation Factor (C): the vegetation quantity on the ground surface has a significant impact on sand / soil erosion by wind. Different vegetation types have different effects of sandstorm prevention. We divide the vegetation into six vegetation types: woodland, shrub, grassland, farmland, bare land and desert, and calculate the C value according to different coefficients and the vegetation coverage.

Surface Roughness Factor (K'): original RWEQ was designed to calculate wind erosion loss at a field scale. Tillage operations modify the soil surface roughness and flatten and bury crop residues *(25)*. When scaled-up to a region, we replaced soil ridge roughness with roughness caused by topography, which we calculate using the Smith-Carson equation.

(iii) Water retention

We estimated the annual water retained in ecosystems using the following model:

$$TQ = \sum_{i=1}^{j} (P_i - R_i - ET_i) \cdot A_i$$

where TQ is total water retention, P_i is precipitation, R_i is storm runoff, ET_i is evapotranspiration and A_i is the area of the ecosystem as defined by land cover.

We acquired rainfall from 726 meteorological stations nationwide and got the national precipitation map through spatial interpolation. Evapotranspiration data was acquired from Chinese Academy of Sciences.

Runoff coefficient values were estimated from >300 publications on surface runoff across a range of land ecosystems. We divide the natural vegetation into six vegetation types: woodland, shrub, grassland, farmland, bare land and desert, and the value of each vegetation coverage factor is calculated according to different coefficients (Table S4):

$$R = P \times \alpha$$

where R is the runoff, P is the precipitation and α is the runoff coefficient.

(iv) Carbon sequestration and storage

Carbon sequestration refers to the carbon sequestered by terrestrial ecosystems which thereby slows down the current rate of atmospheric CO_2 growth *(26)*; storage refers to the carbon remaining in terrestrial ecosystems, possibly over the long term *(27, 28)*. Carbon storage represents not only the result of carbon sequestration *(28)*, but also indicates the importance of restoration or avoidance of deforestation *(29)*. We examined the dynamics of vegetation biomass carbon storage in China's forest, grassland and wetland ecosystems due to reclamation for cropland / restoration from cropland. The amount of carbon sequestered by cropland vegetation is not considered, since cropland vegetation is harvested every year, and the fixed carbon is returned to the atmosphere or enters the soil. The biomass carbon storage of different types of ecosystem (BCS_{in}) was obtained with the following formula:

$$BCS_{in} = \sum_{j=1}^{n} BCD_{ijm} \times AR_i$$

where BCD_{ijm} is the biomass carbon density of ecosystem i in pixel j in year m. Ecosystem i could be forest and shrubland or grassland, and year m could be 2000 or 2015. The unit of BCD_{ijm} is t C/km^2. AR_i (km^2) is the area of each pixel. The BCD_{ijm} is derived with the following formula:

$$BCD_{ijm} = B_{ijm} \times CC_i$$

where B_{ijm} (in t/km^2) is the biomass density of ecosystem i in pixel j in year m. The data comes from the Aerospace Information Research Institute of the Chinese Academy of Sciences. CC_i is the carbon content in the biomass of ecosystem i, which is 0.5 for forest and wetland and 0.45 for grassland *(30, 31)*.

Estimation of wildlife habitat and its relative importance

We selected threatened species in the IUCN Red List (www.iucnredlist.org/) or China's Red List as indicator species, including categories of critically endangered (CR), endangered (EN) and vulnerable (VU) *(32–37)*. The final selected list contains a total number of 1,534 species, including 955 plants, 152 mammals, 127 birds, 177 amphibians and 123 reptiles. Distribution information for plants is from the Scientific Database of China Plant Species *(38)*. Range maps for mammals, amphibians, and

reptiles are from IUCN (www.iucnredlist.org/) and are supplemented using data from Fei et al. *(39)* and Jiang et al. *(40)*. Range maps for birds are from BirdLife International (http://datazone.birdlife.org).

Because the range maps contained unsuitable habitat, we refined the potential habitat for each species based on specific distribution area, elevational range and vegetation, as suggested by Li and Pimm *(41)*. Data on specific distribution areas are from the Institute of Zoology of the Chinese Academy of Sciences and supplemented by recent studies *(35, 40, 42–53)*. Ecological requirements with respect to elevation and vegetation for each species are from the Scientific Database of China Plant Species, the IUCN Red List, BirdLife International and recent scientific studies *(40, 43, 51, 53–55)*. We extracted elevational data using the 90-Meter Digital Elevation Model from the NASA Shuttle Radar Topographic Mission, and vegetation data from the ecosystem maps in 2000 and 2015.

We identified important areas for species conservation by summing up weighted potential habitats for each taxon. To describe the relative importance of different IUCN categories, we gave weights of 3, 2 and 1 to the categories CR, EN and VU, respectively. For each taxon, we normalized the summed values separately to the range of 0–100 using the minimum–maximum normalization method *(56)*, with 100 being the most important and 0 being the least important. The overall importance index map for biodiversity conservation used the maximum value of each pixel among the five taxon layers.

Accuracy Verification

We compiled data from multiple sources to validate ecosystem classification and ecosystem service assessment results from the National Ecosystem Services Assessment process *(1)*. Data from 114,500 field survey points, 5,333 quadrats and 39 long-term ecosystem monitoring stations (CERNweb, http://www.cern.ac.cn) distributed across the country were used for ground validation of ecosystem types, vegetation cover and biomass, among other factors. We also collected meteorological data, historical records of biodiversity, special assessments from several government

agencies (e.g., surveys of soil erosion) and environmental monitoring data (e.g., hydrology) *(1)*. Besides those reported in published work *(1)*, a total of 118,316 independent ground survey samples obtained through random sampling were used for data accuracy verification during the ecosystem classification and ecosystem service parameter acquisition process in the 2010-2015 assessment. The average accuracy of the eight first-level categories of ecosystems was 93.6% and the average accuracy of the 42 third-level categories was 87.7% (Table S5). We also use the field measurement data from 400 sample plots deployed nationwide to verify the vegetation coverage data, finding an average accuracy rate of 87%. The aboveground biomass was verified by field measurement data from 5,093 sample plots distributed nationwide, with an average accuracy of 75%.

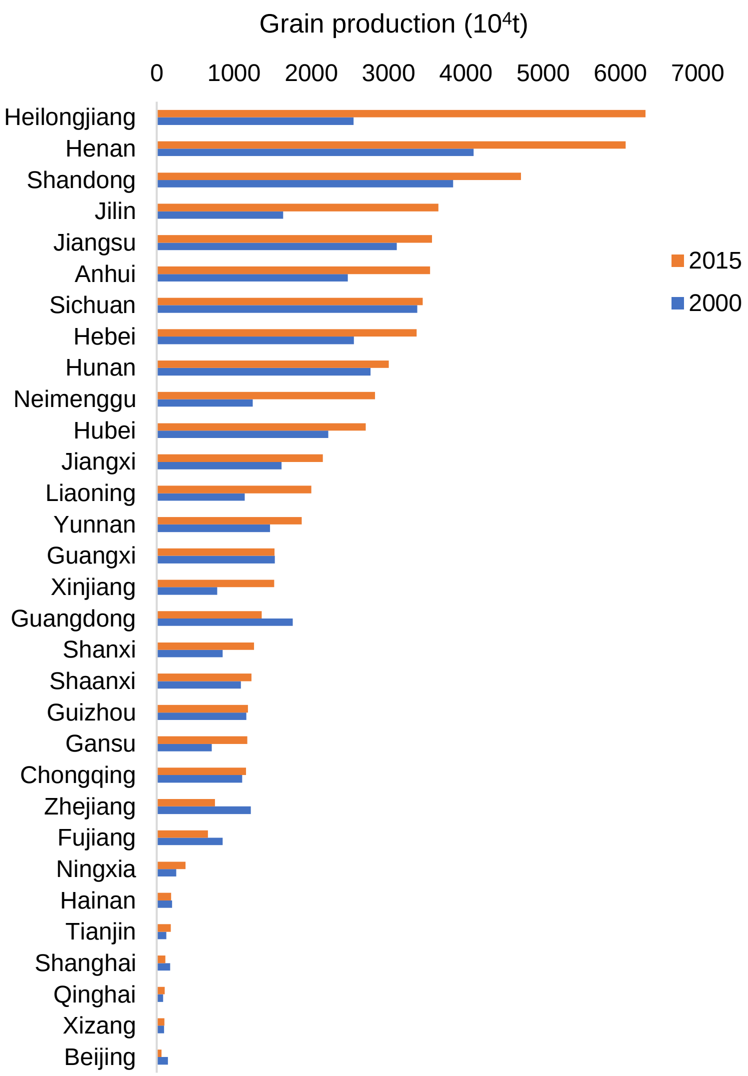

Fig. S1.

Grain production in China's provinces for the years 2000 and 2015.

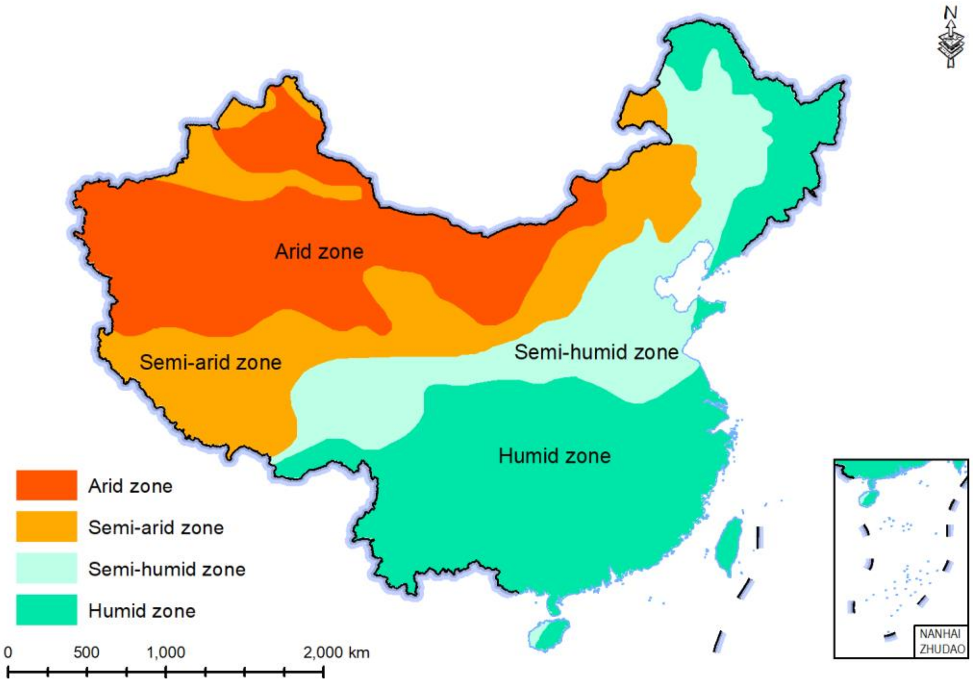

Fig. S2.

The distribution of wet and dry zones across China.

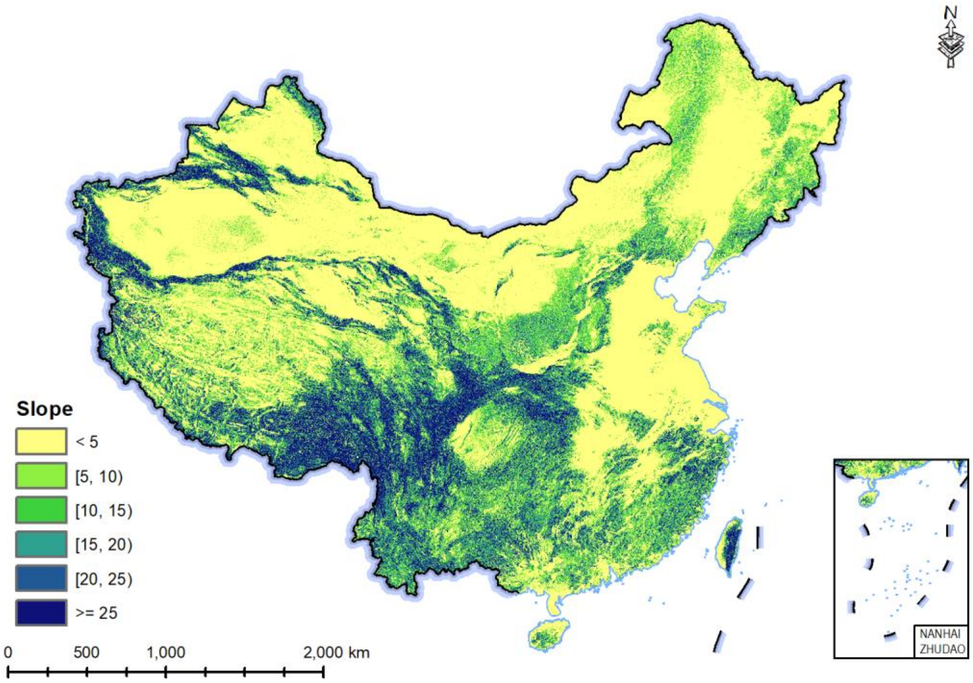

Fig. S3.

Average slope of land across China.

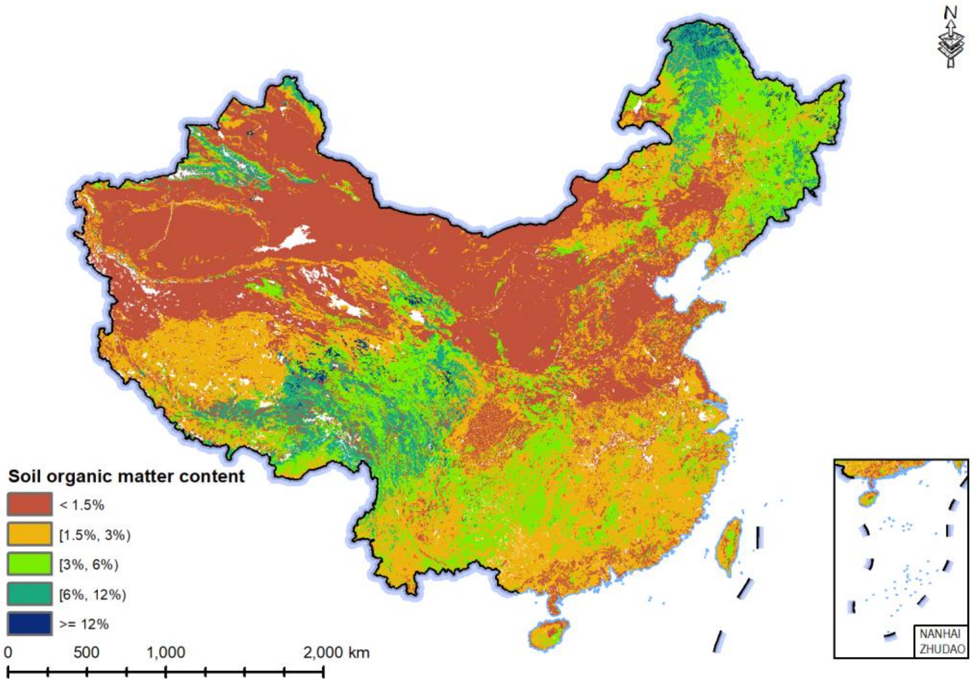

Fig. S4.

The distribution of soil organic matter content across China *(57)*.

Table S1.

The percentages of natural ecosystem importance levels in newly reclaimed farmland.

Importance level of ecosystem services	Area（$10^4 km^2$）	Proportion（%）
Vital	3.43	58.10
Important	1.59	26.82
Moderate	0.43	7.27
General	0.46	7.80

Table S2.

Data sources for ecosystem service evaluation.

Ecosystem Services	Unit	Data Source
Soil retention	t/km^2	Classification map of ecosystems in 2000 and 2015;<br>Vegetation coverage in 2000 and 2015;<br>Digital elevation model (DEM) (from U.S. Geological Survey);<br>Soil properties (from WestDC (http://westdc.westgis.ac.cn/));<br>Rainfall erosivity (from Beijing Normal University)
Water retention	m^3/km^2	Classification map of ecosystems in 2000 and 2015;<br>Precipitation (from China Meteorological Administration);<br>Evapotranspiration (from Institute of Geographic Sciences and Natural Resources Research, CAS)
Sandstorm prevention	t/km^2	Classification map of ecosystems in 2000 and 2015;<br>Vegetation coverage in 2000 and 2015;<br>Soil properties;<br>DEM;<br>Precipitation;<br>Temperature (from China Meteorological Administration);<br>Wind speed (from Institute of Geographic Sciences and Natural Resources Research, CAS);<br>Solar radiation (from WestDC)
Carbon sequestration	gC/m^2	Classification map of ecosystems in 2000 and 2015;<br>Ecosystem biomass in 2000 and 2015

Data on the classification maps of ecosystems, vegetation coverage and ecosystem biomass were taken from Database of China Ecosystem Assessment and Ecological Security (http://www.ecosystem.csdb.cn/); t = metric tons.

Table S3.

Ecosystem classification system based on remote sensing data *(1)*.

I Code	I Class	II Code	II Class	III Code	III Class
1	Forest	11	Broad-leaved forest	111	Evergreen broad-leaved forest
				112	Deciduous broad-leaved forest
		12	Coniferous forest	121	Evergreen coniferous forest
				122	Deciduous coniferous forest
		13	Mixed broadleaf-conifer forest	131	Mixed broadleaf-conifer forest
		14	Sparse forest	141	Sparse forest
2	Shrub	21	Broad-leaved shrub	211	Evergreen broad-leaved shrub
				212	Deciduous broad-leaved shrub
		22	Coniferous shrub	221	Evergreen coniferous shrub
		23	Sparse shrub	231	Sparse shrub
3	Grassland	31	Meadow	311	Meadow
		32	Steppe	321	Steppe
		33	Grass	331	Grass
		34	Sparse grassland	341	Sparse grassland
4	Wetland	41	Wetland	411	Forest wetland
				412	Shrub wetland
				413	Grass wetland
		42	Lake	421	Lake
				422	Reservoir (human-made)
		43	River	431	River
				432	Canal (human-made)
5	Cropland	51	Farmland	511	Paddy land
				512	Dryland
		52	Orchard	521	Tree orchard
				522	Shrub orchard
6	Urban	61	Residential land	611	Residential land
		62	Urban green land	621	Tree green land
				622	Shrub green land
				623	Grass green land

I Code	I Class	II Code	II Class	III Code	III Class
		63	Industrial, mining & transportation land	631	Industrial land
				632	Transportation land
				633	Mining land
7	Desert	71	Desert	711	Desert
				712	Bare rock desert
				713	Bare soil desert
				714	Saline and alkaline land desert
8	Others	81	Glacier and perennial snowfield	811	Glacier and perennial snowfield
		82	Bareland	821	Mosses/lichens
				822	Bare rock
				823	Bare soil
				824	Saline and alkaline land
				825	Sand

Ecosystems with III Code of 111~421 and 431 are natural ecosystems. Ecosystems with III Code of 422, 432~633 are artificial ecosystems.

Table S4.

The mean runoff coefficient value of different natural ecosystem types.

Ecosystem types		Mean coefficient value (%)
Forest	Evergreen broad-leaved forest	2.67
	Deciduous broadleaved forest	1.33
	Evergreen coniferous forest	3.02
	Deciduous coniferous forest	0.88
	Mixed broadleaf-conifer forest	2.29
	Sparse forest	19.20
Shrub	Evergreen broadleaf shrubs	4.26
	Deciduous broad-leaved shrub	4.17
	Evergreen coniferous shrub	4.17
	Sparse shrub	19.2
Grassland	Meadow	8.20
	Steppe	4.78
	Grass	9.37
	Sparse grassland	18.27
Wetland	Wetland	0
Farmland	Arbor garden	9.57
	Shrub garden	7.90
	Paddy field	34.70
	Dry land	49.69

Table S5.

Classification accuracy of first-level categories of natural ecosystems.

First-level categories	Accuracy
Forest	94.43%
Grassland	90.48%
Wetland	91.23%
Cropland	94.74%

References

1. Z. Ouyang, H. Zheng, Y. Xiao, S. Polasky, J. Liu, W. Xu, Q. Wang, L. Zhang, Y. Xiao, E. Rao, L. Jiang, F. Lu, X. Wang, G. Yang, S. Gong, B. Wu, Y. Zeng, W. Yang, G. C. Daily, Improvements in ecosystem services from investments in natural capital. *Science* **352**, 1455-1459 (2016).
2. W. H. Wischmeier, D. D. Smith, *Predicting Rainfall Erosion Losses: A Guide to Conservation Planning* (U.S. Department of Agriculture, Washington, DC, 1978).
3. R. Sharp *et al*., *InVEST +VERSION+ User's Guide* (The Natural Capital Project, Stanford University, University of Minnesota, The Nature Conservancy, and World Wildlife Fund, 2015).
4. P. Kareiva, H. Tallis, T. H. Ricketts, G. C. Daily, S. Polasky, Eds., *Natural Capital: Theory and Practice of Mapping Ecosystem Services* (Oxford Univ Press, New York, 2011).
5. W. Wang, J. Jiao, Quantitative evaluation on factors influencing soil erosion in China. *Bull. Soil Water Conserv.* **16**, 1–20 (1996).
6. S. Q. Yin, W. B. Zhang, Y. Xie, S. H. Liu, F. Liu, Spatial distribution of rainfall erosivity in China based on high-density station network. *Soil Water Conserv. China* **10**, 45–51 (2013).
7. J. R. Williams, K. G. Renard, P. T. Dyke, EPIC - A new method for assessing erosions effect on soil productivity. *J. Soil Water Conserv.* **38**, 381–383 (1983).
8. K. L. Zhang, A. P. Shu, X. L. Xu, Q. K. Yang, B. Yu, Soil erodibility and its estimation for agricultural soils in China. *J. Arid Environ.* **72**, 1002–1011 (2008). doi:10.1016/j.jaridenv.2007.11.018
9. R. Van Remortel, M. Hamilton, R. Hickey, Estimating the LS-factor for RUSLE through iterative slope length processing of digital elevation data within ArcInfo grid. *Cartography* **30**, 27–35 (2001). doi:10.1080/00690805.2001.9714133
10. D. K. McCool, G. O. George, M. Freckleton, C. L. Douglas, R. I. Papendick, Topographic effect on erosion from cropland in the northwestern wheat region. *Trans. Am. Soc. Agric. Engin.* **36**, 1067–1071 (1993). doi:10.13031/2013.28435
11. B. Y. Liu, M. A. Nearing, L. M. Risse; Slope gradient effects on soil loss for steep slopes. *Trans. Am. Soc. Agric. Engin.* **37**, 1835–1840 (1994). doi:10.13031/2013.28273
12. E. M. Rao, Z. Ouyang, X. Yu, Y. Xiao, Spatial patterns and impacts of soil conservation service in China. *Geomorphology* **207**, 64–70 (2014). doi:10.1016/j.geomorph.2013.10.027
13. H. J. Carter, D. L. Eslinger, *Nonpoint Source Pollution and Erosion Comparison Tool (N-SPECT) Technical Guide* (National Oceanic and Atmospheric Administration Coastal Services Center, Charleston, SC, 2004).
14. B. Liu, S. Liu, S. Zheng, Soil conservation and coefficient of soil conservation of crops. *Res. Soil Water Conserv.* **6**, 32–36 (1999).
15. H. B. Wei, R. Li, Q. K. Yang, Research advances of vegetation effect on soil and water conservation in China. *Acta Phytoecol. Sin.* **26**, 489–496 (2002).
16. D. W. Fryrear, A. Saleh, J. D. Bilbro, H. M. Schomberg, J. E. Stout, T. M. Zobeck,

Revised Wind Erosion Equation (RWEQ) (Tech. Bull. 1, Wind Erosion and Water Conservation Research Unit, USDA-ARS, Southern Plains Area Cropping Systems Research Laboratory, Lubbock, TX, 1998).
17. E. L. Skidmore, J. Tatarko, Stochastic wind simulation for erosion modeling. *Trans. Am. Soc. Agric. Engin.* **33**, 1893–1899 (1990). doi:10.13031/2013.31555
18. E. L. Skidmore, J. Tatarko, L. E. Wagner, in *Proceedings of a Workshop on Climate and Weather Research*, C. W. Richardson, V. A. Ferreira, P. C. Doraiswamy, Eds., Denver, CO, 17 to 19 July 1995 (U.S. Department of Agriculture, Washington, DC, 1996).
19. R. A. Bagnold, *The Physics of Blown Sand and Desert Dunes* (Methuen, London, 1943)
20. A. W. Zingg, Some characteristics of aeolian sand movement by saltation process. *Editions Centre National Recherche Scientifique* **7**, 197–208 (1953).
21. W. S. Chepil, A compact rotary sieve and the importance of dry sieving in physical soil analysis. *Soil Sci. Soc. Am. Proc.* **26**, 4–6 (1962). doi:10.2136/sssaj1962.03615995002600010002x
22. D. W. Fryear, C. A. Krammes, D. L. Williamson, T. M. Zobeck, Computing the wind erodible fraction of soils. *J. Soil Water Conserv.* **49**, 183–188 (1994).
23. T. M. Zobeck, Abrasion of crusted soils: Influence of abrader flux and soil properties. *Soil Sci. Soc. Am. J.* **55**, 1091–1097 (1991). doi:10.2136/sssaj1991.03615995005500040033x
24. L. J. Hagen, E. L. Skidmore, A. Saleh, Wind erosion: Prediction of aggregate abrasion coefficients. *Trans. Am. Soc. Agric. Engin.* **35**, 1847–1850 (1992). doi:10.13031/2013.28805
25. R. G. Nelson, L. E. Wagner, K. Stueve, in *Proc. ASAE Winter Meeting*, Chicago, IL, 14 to 17 December 1993, Paper 932539 (1993).
26. S. Piao, J. Fang, P. Ciais, P. Peylin, Y. Huang, S. Sitch, T. Wang, The carbon balance of terrestrial ecosystems in China. *Nature* **458**, 1009–1013 (2009). Medline doi:10.1038/nature07944
27. S. L. Lewis, G. Lopez-Gonzalez, B. Sonké, K. Affum-Baffoe, T. R. Baker, L. O. Ojo, O. L. Phillips, J. M. Reitsma, L. White, J. A. Comiskey, M.-N. Djuikouo K, C. E. N. Ewango, T. R. Feldpausch, A. C. Hamilton, M. Gloor, T. Hart, A. Hladik, J. Lloyd, J. C. Lovett, J.-R. Makana, Y. Malhi, F. M. Mbago, H. J. Ndangalasi, J. Peacock, K. S.-H. Peh, D. Sheil, T. Sunderland, M. D. Swaine, J. Taplin, D. Taylor, S. C. Thomas, R. Votere, H. Wöll, Increasing carbon storage in intact African tropical forests. *Nature* **457**, 1003–1006 (2009). Medline doi:10.1038/nature07771
28. H. Keith, B. G. Mackey, D. B. Lindenmayer, Re-evaluation of forest biomass carbon stocks and lessons from the world's most carbon-dense forests. *Proc. Natl. Acad. Sci. U.S.A.* **106**, 11635–11640 (2009). Medline doi:10.1073/pnas.0901970106
29. L. Mandle, H. Tallis, L. Sotomayor, A. Vogl, Tracking ecosystem service redistribution from road development and mitigation in the Peruvian Amazon. *Front. Ecol. Environ* **13**, 309–315 (2015). doi:10.1890/140337
30. P. Q. Chen, X. K. Wang, L. M. Wang, *Carbon Budget and Its Sink Promotion of*

Terrestrial Ecosystem in China (Science Press, Beijing, 2008).
31. J. Fang, Y. Yang, W. Ma, A. Mohammat, H. Shen, Ecosystem carbon stocks and their changes in China's grasslands. *Sci. China Life Sci.* **53**, 757–765 (2010). Medline doi:10.1007/s11427-010-4029-x
32. Ministry of Ecology and Environment of the People's Republic of China and Chinese Academy of Sciences (2015) Redlist of China's biodiversity-vertebrate. Available at www.mee.gov.cn/gkml/hbb/bgg/201505/t20150525_302233.htm. Accessed May 15, 2022.
33. Ministry of Ecology and Environment of the People's Republic of China and Chinese Academy of Sciences (2013) Redlist of China's biodiversity-higher plants. Available at www.mee.gov.cn/gkml/hbb/bgg/201309/t20130912_260061.htm. Accessed May 15, 2022.
34. Z. Jiang, et al, Evaluating the status of China's mammals and analyzing their causes of endangerment through the red list assessment. *Biodiv Sci.* **24**, 552–567 (2016).
35. J. Jiang, et al, Assessing the threat status of amphibians in China. *Biodiv Sci.* **24**, 588–597 (2016).
36. B. Cai, J. Li, Y. Chen, Y. Wang, Exploring the status and causes of China's threatened reptiles through the red list assessment. *Biodiv Sci.* **24**, 578–587 (2016).
37. Y. Zhang, et al, Assessment of red list of birds in China. *Biodiv Sci.* **24**, 568–577 (2016).
38. Kunming Institute of Botany Chinese Academy of Sciences (2016) Scientific Database of China Plant Species. Available at db.kib.ac.cn/eflora/Default.aspx. Accessed January 15, 2017.
39. L. Fei, C. Ye, J. Jiang, Colored Atlas of Chinese Amphibians and Their Distributions (Sichuan Publishing House of Science and Technology, Chengdu, 2012).
40. Z. Jiang, et al, China's Mammals Diversity and Geographic Distribution (Science Press, Beijing, 2015).
41. B. V. Li, S. L. Pimm, China's endemic vertebrates sheltering under the protective umbrella of the giant panda. *Conserv. Biol.* **30**, 329–339 (2016).
42. H. Ai, The geographical distribution and ecological research status of Budorcas Taxicolor in China. *Sichuan Dong Wu* **22**,14–17 (2003).
43. D. Li, Geographical distribution and protection status of threatened species of Anatidae in China. Master thesis (Beijing Forestry University, Beijing, 2014). Chinese.
44. W. Liu, The distribution and present status of wild yak. *Xizang Technology* **11**, 17–23 (2003).
45. C. Wang, et al, The current population and distribution of wild crested ibis Nipponia nippon. *Dongwuxue Zazhi* **49**, 666–671(2014).
46. C. Wang, The distribution and conservation strategy of Moschus Anhuiensis. *Anhui Forestry Science and Technology* (3):65–66 (2010).
47. M. Wu, The relationship between the distribution of endemic, rare and endangered bird and climatic factors in China. Master thesis (Southwest University, Chongqing, 2011). Chinese.

48. M. Wu, N. Wang, D. Hui, X. Wang, Status and research progress of Forest Musk Deer protection. *Journal of Chongqing University of Technology* **25**, 34–39 (2011).
49. W. Wu, et al, Impact of climate change on distribution of breeding sites of redcrowned crane in China. *J Ecol Rural Environ* **28**, 243–248 (2012).
50. Y. Xue, et al, Research and conservation of wild camel: Present status and future prospects. *Sichuan Dong Wu* **33**, 476–480 (2014).
51. M. Yao, Study on the distribution pattern of amphibia in China. Master thesis (Central South University of Forestry & Technology, Zhuzhou, 2014). Chinese.
52. T. Zhou, Distribution of Chinese chelonians. *Sichuan Dong Wu* **25**, 272–276 (2006).
53. Y. Zhou, B. Yu, X. Wu, J. Nie, Present status of protection on Chinese alligator's population and habitat. *Dongwuxue Zazhi* **47**, 133–136 (2012).
54. Y. Huang, Y. Wang, Progress in the biogeography of amphibian and reptile in Hainan Island. *Sichuan Dong Wu* **30**, 304–309 (2011).
55. J. Jiang, The status of resource and conservation of great bustard in China. Master thesis (Northeast Forestry University, Harbin, 2004). Chinese.
56. W. Yang, T. Dietz, D. B. Kramer, Z. Ouyang, J. Liu, An integrated approach to understanding the linkages between ecosystem services and human well-being. *Ecosystem Health and Sustainability* **1**, (2015). doi: 10.1890/EHS15-0001.1
57. Dai, Y., Shangguan, W. Dataset of soil properties for land surface modeling over China. A Big Earth Data Platform for Three Poles. (2019). https://doi.org/10.11888/Soil.tpdc.270281. https://cstr.cn/18406.11.Soil.tpdc.270281.
